# The Inhibitory Effect of PIK-75 on Inflammatory Mediator Response Induced by Hydrogen Peroxide in Feline Esophageal Epithelial Cells

**DOI:** 10.1155/2014/178049

**Published:** 2014-09-07

**Authors:** Jun Yeong Jeong, Yeon Joo Lee, Jeong Hoon Han, Sun Young Park, Kwang Woo Hwang, Uy Dong Sohn

**Affiliations:** ^1^Signaling and Pharmacological Activity Research Lab, College of Pharmacy, Chung-Ang University, Seoul 156-756, Republic of Korea; ^2^Host Defense Modulation Lab, College of Pharmacy, Chung-Ang University, Seoul 156-756, Republic of Korea

## Abstract

Isoform-selective inhibitors of phosphoinositide 3-kinase (PI3K) activation have an anti-inflammatory effect by reducing proinflammatory cytokines. Cultured feline esophageal epithelial cells (EEC) of passages 3~4 were treated with hydrogen peroxide and PIK-75. The cell viability was measured by a MTT incorporation assay. The distribution of PI3K isoforms, p-Akt, IL-1*β*, and IL-8 was inferred from Western blots. The release of IL-6 was determined by ELISA. The cell morphology was not considerably different from nontreated cells if the cells were pretreated with PIK-75 and treated with 300 *μ*M hydrogen peroxide. The density of p110α of PI3K was increased, but that of other types was not affected after the treatment with hydrogen peroxide. The density of p-Akt, when the cells were exposed to PIK-75 and hydrogen peroxide, was diminished dose dependently more than that of hydrogen peroxide treatment only. The decrease of p-Akt showed an inhibition of PI3K by PIK-75. PIK-75 dose dependently reduced the expression of IL-1*β*, IL-8, and the level of IL-6 compared with hydrogen peroxide treatment only. These results suggest evidence that p110α mediates esophageal inflammation and that PIK-75 has an anti-inflammatory effect by reducing proinflammatory cytokines on feline esophageal epithelial cultured cells.

## 1. Introduction

Phosphoinositide 3-kinase (PI3K) is a family of lipid kinases that phosphorylate the 3′-hydroxyl group of phosphoinositides and phosphatidylinositol [1]. The lipid products of PI3K reactions create binding sites for specific, lipid-binding domains on many intracellular signaling proteins [2]. Therefore, it contributes to a wide range of cellular responses to extracellular signals and in various signal transduction pathways [3, 4].

PI3K is revealed to play an essential role in the regulation of intestinal epithelial proliferation, survival, and wound healing [5]. Its pathways have been shown to be associated with many cancers such as breast, colorectal, and esophageal cancer [6, 7]. They are also involved in the development of inflammatory diseases such as arthritis and atherosclerosis [8, 9]. PI3K is an important mediator in the signaling cascade leading to the initiation of the inflammatory response including an enhanced release of proinflammatory cytokines [10, 11].

PI3K is on the basis of structural and functional aspects divided into three classes (I, II, and III). While the involvement of class II and class III PI3K in inflammation has not yet been defined, class I enzymes are considered as an important element in many steps of the inflammatory reaction [12, 13]. Class I PI3K is consisting of a complex formed by catalytic subunits (C) and regulatory subunits (R). Three R (p85α, p85*β*, and p55*γ*) and four C (p110α, p110*β*, p110*γ*, and p110*δ*) isoforms are known [4, 14–17]. It is well established that PI3K isoforms could be distinctively expressed according to the cell type [4, 18]. Targeting PI3K is compelling therapeutic approach and some inhibitors have been clinically tested to be developed as potent drugs [19, 20]. Nonselective PI3K inhibitors, wortmannin, and LY294002 are unsuitable for a therapeutic approach because its lack of selectivity may produce toxicity issues [21].

Many studies have established that an increased production of proinflammatory cytokines is critical for the pathogenesis of inflammatory disorders [22]. Various inflammation inducers such as TNF-α enhance the secretion of IL-1*β*, IL-6, and IL-8 [23, 24]. IL-8 is a potent chemokine implicated in the recruitment of neutrophils during inflammation. The levels of IL-8 have significantly increased in the gastroesophageal reflux disease (GERD) [25, 26]. Also the esophageal epithelium is believed to be the major source of IL-8 production [27].

Hydrogen peroxide is one type of the reactive oxygen species (ROS) [28]. Increased ROS in mucosa have been implicated as a trigger of gastrointestinal diseases, including inflammatory disorders and acid-related peptic diseases [29]. Hydrogen peroxide is a well-known inducer of esophageal epithelial inflammation and is elevated in GERD [30, 31].

In the present study, we investigated the expression of PI3K isoforms and the anti-inflammatory potential effect of PIK-75 as a selective p110α inhibitor [32] in feline EECs.

## 2. Materials and Methods

### 2.1. Reagents

Hydrogen peroxide, 4-(2-hydroxyethyl)-1-piperazine-N′-2-ethane sulfonic acid (HEPES), leupeptin, aprotinin, bovine serum albumin (BSA), *β*-mercaptoethanol, ethylene glycol-bis-(*β*-aminoethylether)-N,N,N′,N′-tetraacetic acid (EGTA), phenylmethylsulfonyl fluoride (PMSF), ethylenediamine tetra acetic acid (EDTA), thiazolyl blue tetrazolium bromide, and Hank's balanced salt solution-modified (HBSS) were purchased from Sigma Chemical Co. (St. Louis, MO, USA). Fetal bovine serum (FBS), antibiotic-antimycotic (penicillin, streptomycin, and amphotericin B), and trypsin-EDTA were purchased from Invitrogen (Grand Island, NY, USA) Tris-buffered saline (TBS), Dulbecco's modified Eagle's medium (DMEM), and phosphate-buffered saline (PBS) were purchased from Welgene Inc. (Daegu, South Korea). PIK-75 was purchased from Cayman (Michigan, CA, USA). p-Akt and t-Akt antibodies were purchased from Cell Signaling Technology (Beverly, MA, USA). IL-1*β* antibody was purchased from Santa Cruz Biotechnology (Santa Cruz, CA, USA). IL-8 antibody was purchased from Bioworld (Dublin, OH, USA). Goat anti-rabbit IgG-HRP, rabbit anti-goat IgG-HRP, and goat anti-mouse IgG-HRP were purchased from Bethyl (Montgomery, TX, USA). Rainbow prestained molecular weight marker was purchased from Amersham (Arlington Heights, IL, USA). Ammonium persulfate, N,N,N′,N′-tetramethylethylenediamine (TEMED), and enhanced chemiluminescence (ECL) agents were purchased from PerkinElmer Life Sciences (Boston, MA, USA). Nitrocellulose (NC) membrane, Tris/Glycine/SDS buffer, Tris/Glycine buffer, and Restore TM Western Blot Stripping Buffer were purchased from Thermo (Rockford, IL, USA); IL-6 ELISA kit was purchased from Enzo (Farmingdale, NY, USA) and the 30% acrylamide/bis solution was purchased from Bio-Rad (Richmond, CA, USA).

### 2.2. Preparation of Feline Esophageal Epithelial Tissue Squares

All animal experiments were performed in accordance with the guidelines of the Institutional Animal Care and Use Committee of the Institute for Molecules-Based New Drug Development. Adult cats of either sex weighing between 2.5 and 3.5 kg were anesthetized with Zoletil 50 (12.5 mg/0.25 mL/kg) before the abdomen was opened with a midline incision. The esophagus was excised, washed, and maintained in Krebs buffer composed of 116.6 mM NaCl, 1.2 mM NaH_2_PO_4_, 21.9 mM NaHCO_3_, 3.4 mM KCl, 5.4 mM glucose, 2.5 mM CaCl_2_, and 1.2 mM MgCl_2_. The esophagus was opened along the lesser curvature. The location of the squamocolumnar junction was identified. The mucosa was peeled off. The submucosal connective tissues were then removed by microspring scissors. The mucosa from esophagus was sliced into 0.5 mm thick sections with a Stadie Riggs tissue slicer (Thomas Scientific Apparatus, Philadelphia, PA, USA). The last slices were cut into 2 mm × 2 mm tissue squares with scissors.

### 2.3. Cultures of Feline EECs

The sliced tissue was placed into DMEM supplemented with 10% FBS containing 100 U/mL penicillin, 0.1 mg/mL streptomycin, and 0.25 *μ*g/mL amphotericin B and incubated in a humidified atmosphere of 5% CO_2_ and 95% air at 37°C. After 10 days, the medium was exchanged with fresh DMEM containing 10% FBS. After reaching confluence, the cells were detached using 1% trypsin-EDTA in HBSS with bicarbonate. The cells were then counted, seeded at 2 × 10^5^ cells/mL on 100 mm culture dishes, and maintained in DMEM containing 10% FBS. The medium was changed every 48 hours until the cells reached confluence. Experiments were performed on cells at passage 3 or 4.

### 2.4. Measurements of Cell Viability

The cell viability was determined by the conventional 3-(4,5-dimethylthiazol-2-yl)-2,5-diphenyltetrazolium bromide (MTT) reduction assay. In this assay, viable cells convert MTT to insoluble blue formazan crystals via the mitochondrial respiratory chain enzyme succinate dehydrogenase. The cells were plated at a density of 1 × 10^5^/well on 6-well plates and maintained in DMEM containing 10% FBS. When the cells were made quiescent at confluence by incubation, the media was changed with serum-free DMEM for 24 hours to arrest cell growth and silence gene activity, followed by treatment with each indicated agent for the indicated time periods. After incubation, the cells were rapidly washed twice with ice-cold PBS and incubated with MTT solution (final concentration, 5 mg/mL) for 4 hours at 37°C. Then, the supernatant was removed and the formazan crystals were dissolved with DMSO. Absorbance was monitored at 570 nm with a microplate reader (Molecular Devices, Sunnyvale, CA, USA).

### 2.5. Preparation of Cell Extracts

When the cells reached confluence, they were serum starved by incubation in serum-free DMEM for 24 hours. The cells were then stimulated with each compound for the indicated time periods or at the indicated concentrations. After incubation, the cells were rapidly washed twice with ice-cold PBS and lysed with ice-cold lysis buffer (20 mM Tris-HCl (pH 7.4), 0.5 mM EDTA, 0.5 mM EGTA, 1% (w/v) Triton X-100, 0.01% (w/v) SDS, 10 g/mL leupeptin, 10 *μ*g/mL aprotinin, 1 mM PMSF, and 0.7 *μ*g/mL *β*-mercaptoethanol) for 5 minutes. The lysates were scraped with a cell scraper and collected in Eppendorf tubes. They were then sonicated (6 seconds, 3x) and centrifuged to remove cellular debris for 10 minutes with 13,000 rpm at 4°C; the supernatants were collected and stored at −70°C for protein assay and Western blot analysis.

### 2.6. Protein Determination

The protein concentration of the supernatant in each reaction vial was measured spectrophotometrically using the Bio-Rad assay (Bio-Rad Chemical Division, Richmond, CA, USA). Absorption was monitored at 595 nm.

### 2.7. Western Blot Analysis

Equal amounts of protein from each sample were subjected to electrophoresis on a 10% SDS-polyacrylamide gel and transferred to a NC membrane using the Power Pac 1000 (Bio-Rad, Melville, NY, USA) power supply. To block any nonspecific binding, the NC membrane was incubated in 5% nonfat dry milk or 3% BSA in TBS for 60 minutes followed by three rinses in milk-free TBS. The membranes were with shaking with primary antibodies raised against p-Akt, IL-1*β*, and IL-8 incubated overnight at 4°C, followed by three washes with TBS containing 0.1% Tween 20. This was followed by 60-minute incubation in horseradish peroxidase-conjugated secondary antibody. Immunoreactive proteins were detected with ECL agent. Molecular masses were estimated by comparison with a prestained molecular mass marker. The same blots were subsequently stripped with Western blot stripping buffer and reprobed with actin and t-Akt antibodies to confirm the uniformity of protein loading. The results were analyzed by Quantity One analysis software (Bio-Rad Chemical Division, Richmond, CA, USA). The percentage of Akt activation or IL-1*β* and IL-8 expression was calculated as the ratio of phosphorylated Akt to total Akt or IL-1*β* and IL-8 to actin.

### 2.8. Measurements of IL-6 Release from EECs

The cells were cultured in 100 mm culture dishes. All cells were pretreated with each indicated agent for the indicated time. EECs were then stimulated with hydrogen peroxide. The medium was collected, centrifuged, and stored at −70°C until assay. The levels of IL-6 released into the culture medium were quantified using an IL-6 ELISA kit. Assays were performed according to the manufacturer's instructions.

### 2.9. Data Analysis

Differences among the groups were analyzed using one-way ANOVA and Student's *t*-test. Data are expressed as the means ± S.E.M. of 3~6 experiments and differences between groups were considered significant at *P* < 0.05.

## 3. Results

### 3.1. Hydrogen Peroxide Induces the Cytotoxicity Effect in Cultured EECs

**Figure 1:**
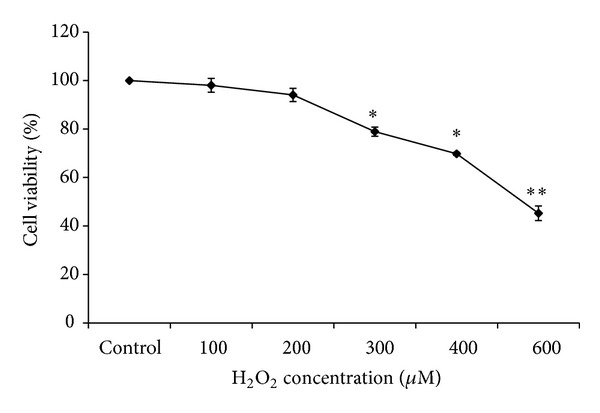
Effect of H_2_O_2_ on the cell viability of feline EECs. Serum-starved EECs were incubated with H_2_O_2 _for 24 hours at the indicated concentration. The cell viability was estimated using MTT assay. Data are expressed as means ± S.E of three experiments (Student's* t*-test; *P < 0.05 versus control; **P < 0.001 versus control).

MTT assays were performed in cultured EECs to investigate the cytotoxic effect of hydrogen peroxide. The cells were incubated with hydrogen peroxide at the indicated concentration for 24 hours and then cell viability was measured using the MTT assay (Figure 1). The cell viability was decreased by 300 *μ*M hydrogen peroxide in a dose-dependent manner and its decrease was apparent at a higher concentration of H_2_O_2_. The viability of cells exposed to 600 *μ*M hydrogen peroxide was reduced to 40% when compared to control. The number of cells was decreased after hydrogen peroxide treatment.

### 3.2. Expression of IL-1*β* and IL-8 Is Increased after Hydrogen Peroxide Treatment

**Figure 2:**
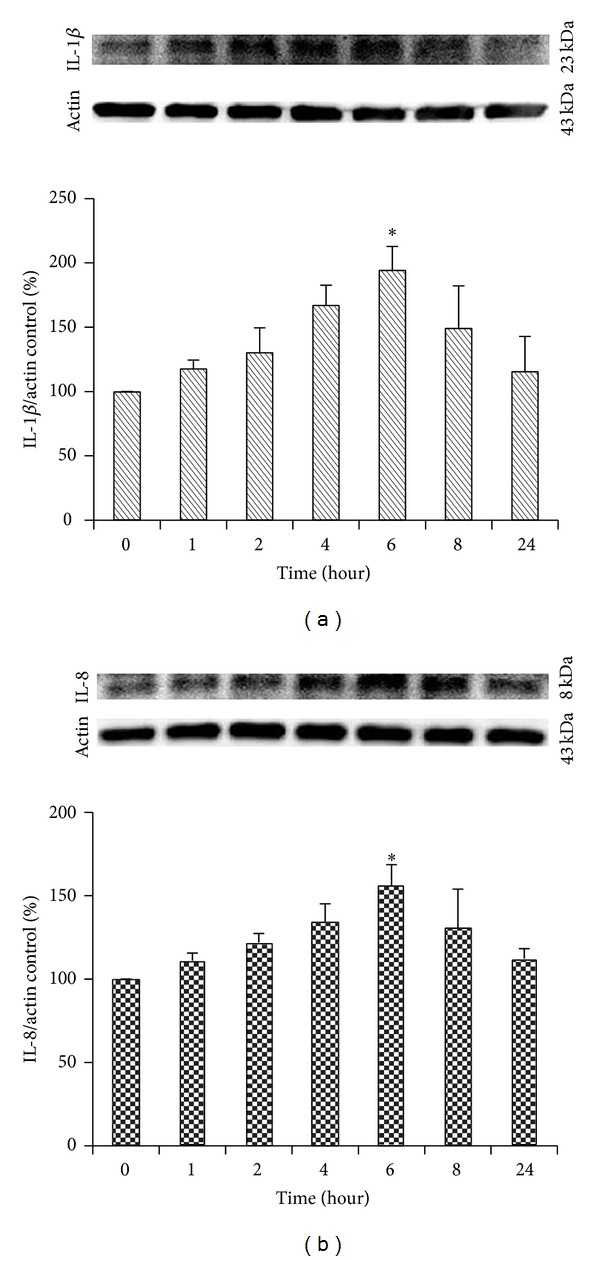
Effect of H_2_O_2_ on the expression of IL-1*β* and IL-8 in feline EECs. The time course of cytokines expression in feline EECs. Feline EECs were exposed to 300 *μ*M H_2_O_2_. (a) Representative Western blot analyses of IL-1*β* expressed in feline EECs (*n* = 3). Actin expression was used as a loading control for normalization. (b) Representative Western blot analyses of IL-8 expressed in feline EECs (*n* = 3). Actin expression was used as a loading control for normalization. Data are expressed as means ± S.E of three experiments (Student's* t*-test; *P < 0.05 versus control).

Serum-starved cells were exposed to 300 *μ*M hydrogen peroxide at the indicated time periods to examine whether hydrogen peroxide induces IL-1*β* and IL-8 expression in cultured EECs. Then IL-1*β* and IL-8 expression was measured by Western blot (Figure 2). 300 *μ*M of hydrogen peroxide which made the maximal expression of 5-LOX was used for inflammation induction [33]. Hydrogen peroxide induced the increased expression of IL-1*β* and IL-8 with a maximal reach at 6 hours. A longer stimulation with hydrogen peroxide reduced the IL-1*β* and IL-8 expression only slightly.

### 3.3. PI3K Subunits Isoforms Are Differentially Expressed in EECs

**Figure 3:**
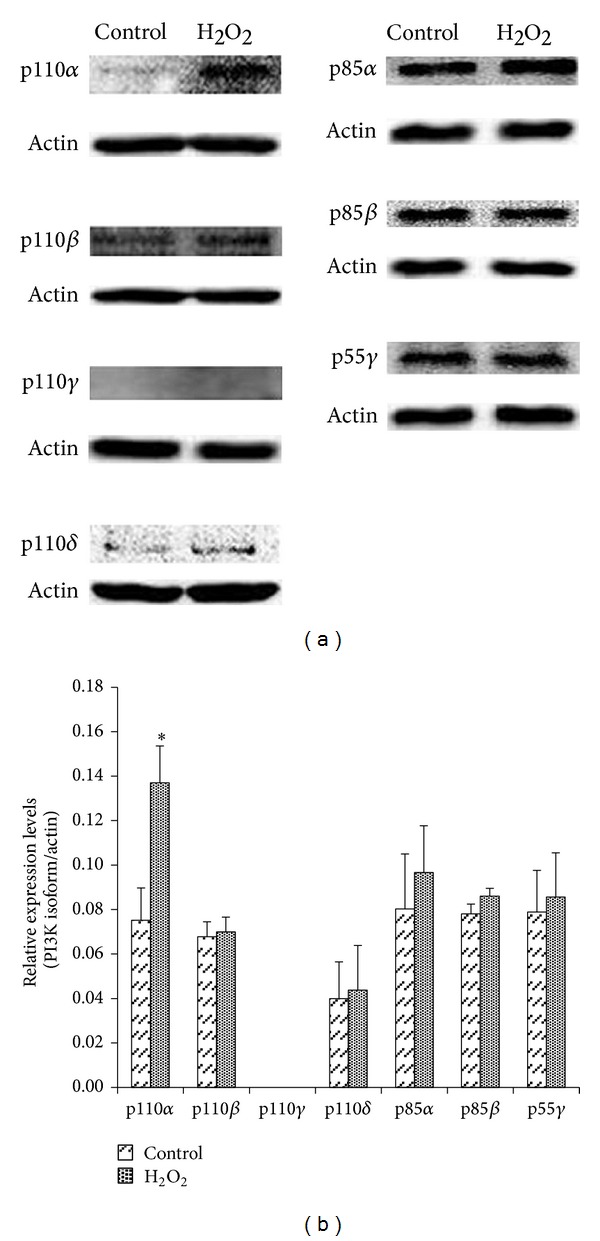
Comparison of PI3K isoforms expressions in feline EECs after treatment with H_2_O_2_. (a) Representative (*n* ≥ 3) Western blot analyses of the expression of the known class PI3K C (p110α, p110*β*, p110*γ*, and p110*δ*) and R (p85α, p85*β*, and p55*γ*) isoforms, using isoform-specific antibodies. Feline EECs were exposed to 300 *μ*M H_2_O_2_ for 6 hours. Actin expression was used as a loading control for normalization. (b) The Western blot bands were quantified in order to establish the relative expression levels for each analyzed isoform. Data are expressed as means ± S.E of three experiments (Student's* t*-test; *P < 0.05 versus control).

The expression profile of class I PI3K R and C isoforms in feline EECs was established (Figure 3). The verification of protein expression by Western blot confirmed that p110α, p85α, p85*β*, and p55*γ* are indeed predominantly expressed and that p110*β*, p110*γ*, and p110*δ* are weakly expressed when the cells were untreated. After the treatment with 300*μ*M hydrogen peroxide for 6 hours the expression of p110α was significantly increased while the expression of p110*β*, p110*γ*, p85α, p85*β*, and p55*γ* was little changed only and slightly increased after the treatment with hydrogen peroxide.

### 3.4. PIK-75 Causes Little Change in the Cell Viability and the Morphology of EECs after Hydrogen Peroxide Stimulation

**Figure 4:**
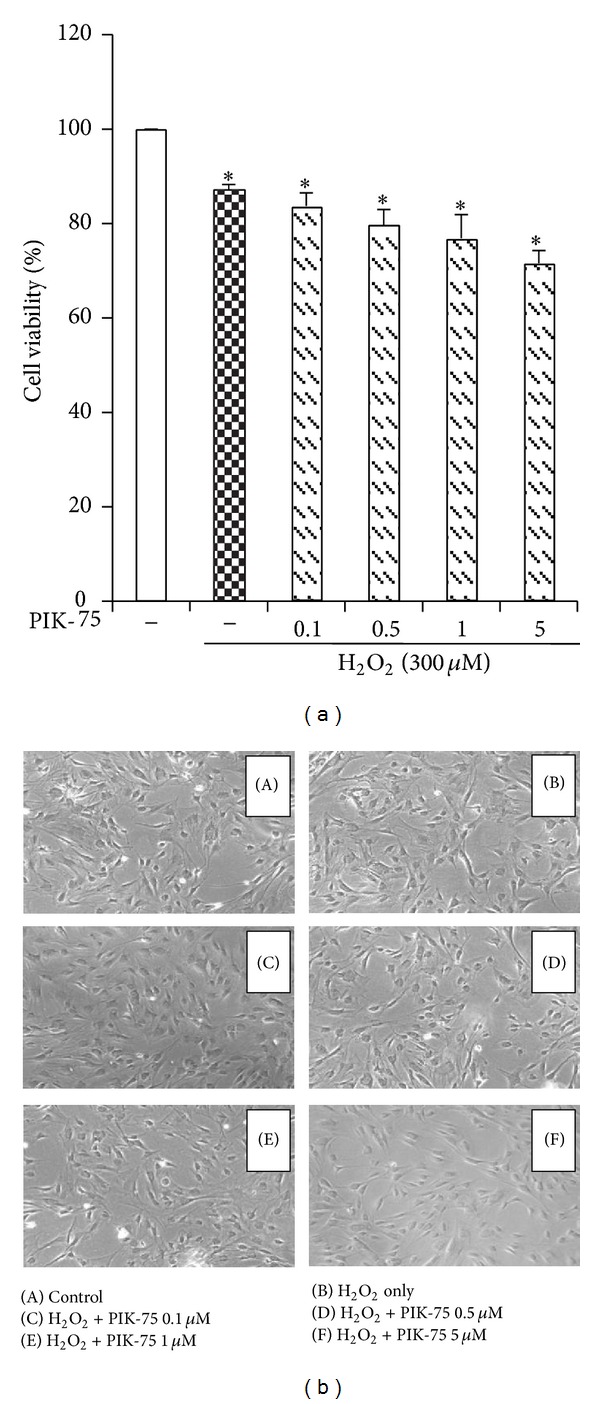
The cell viability and the morphology of H_2_O_2_-treated EECs. Serum-starved EECs were preincubated in the presence of PIK-75 for 1 hour at the indicated concentration. EECs were then stimulated with 300 *μ*M H_2_O_2_ for 6 hours. (a) The cell viability was measured using MTT assay. Data are expressed as means ± S.E of three experiments (Student's* t*-test; *P < 0.05 versus control). (b) The morphologic changes of EECs were observed. Magnification: 100x.

MTT assay had been performed and the morphology of EECs was observed to identify the cell viability and the morphologic changes after the treatment of PIK-75 (Figure 4). Feline EECs were pretreated with PIK-75 at the indicated concentrations (0.1, 0.5, 1, and 5 *μ*M) for 1 hour and treated with 300 *μ*M hydrogen peroxide for 6 hours. Treatment with PIK-75 for 1 hour did not cause strong changes of cell viability and morphology in EECs.

### 3.5. Hydrogen Peroxide-Induced Phosphorylation of Akt Is Reduced by PIK-75 Treatment

**Figure 5:**
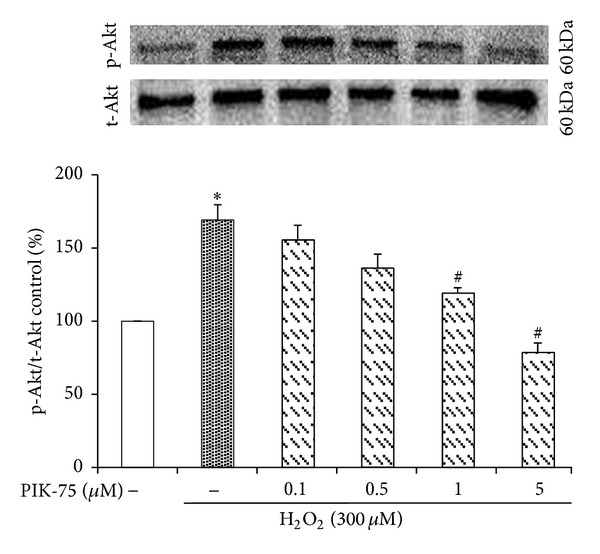
Effect of PIK-75 on p-Akt activation in feline EECs stimulated by H_2_O_2_. Representative Western blot analyses of p-Akt expressed in feline EECs (*n* = 4). EECs were pretreated with PIK-75 at different concentrations, followed by the stimulation with H_2_O_2 _(300 *μ*M, 30 minutes). t-Akt expression was used as a reference. Data are expressed as means ± S.E of four experiments (Student's* t*-test; *P < 0.05 versus control, ^#^ *P* < 0.05 versus H_2_O_2_ alone).

Akt as a major downstream effector of PI3K was examined to determine the effect of PIK-75-mediated PI3K inhibition on downstream signaling events (Figure 5). The cells were treated with 300 *μ*M H_2_O_2_ for 30 minutes to investigate the phosphorylation of Akt. The Western blot analysis of the lysates from H_2_O_2_-stimulated EECs showed a prominent upregulation in the phosphorylation status of Akt compared with lysates from naïve cells. More importantly, the pretreatment of EECs with PIK-75 inhibited the H_2_O_2_-induced phosphorylation of Akt. PIK-75 inhibited p-Akt activation in a dose-dependent manner.

### 3.6. Hydrogen Peroxide-Induced IL-1*β* Expression Is Decreased by PIK-75 Treatment

**Figure 6:**
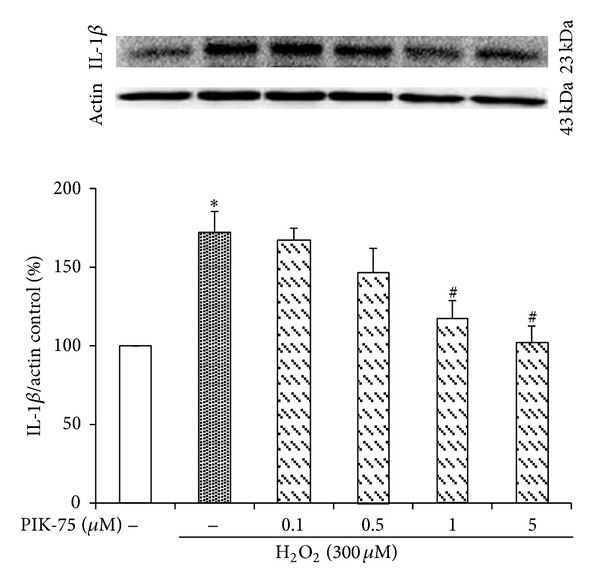
Effect of PIK-75 on IL-1*β* expression in feline EECs stimulated by H_2_O_2_. Serum-starved EECs were preincubated in the presence of PIK-75. EECs were then stimulated with H_2_O_2_ (300 *μ*M, 6 hours). The expression of IL-1*β* was estimated by Western blot analysis. Data are expressed as means ± S.E of four experiments (Student's* t*-test; *P < 0.05 versus control, ^#^ *P* < 0.05 versus H_2_O_2_ alone).

The cells were pretreated with PIK-75 at the indicated concentrations and exposed to 300 *μ*M hydrogen peroxide and then IL-1*β* expression was measured by Western blot to examine whether PIK-75 downregulates IL-1*β* expression in cultured EECs (Figure 6). The expression of IL-1*β* was upregulated when treated with 300 *μ*M hydrogen peroxide for 6 hours. The cells were pretreated with different doses of PIK-75 (0.1, 0.5, 1, and 5 *μ*M) for 1 hour to examine the effect of PIK-75 on H_2_O_2_-induced IL-1*β* expression. PIK-75 inhibited the IL-1*β* expression in a dose-dependent manner.

### 3.7. Hydrogen Peroxide-Induced IL-8 Expression Is Decreased by PIK-75 Treatment

**Figure 7:**
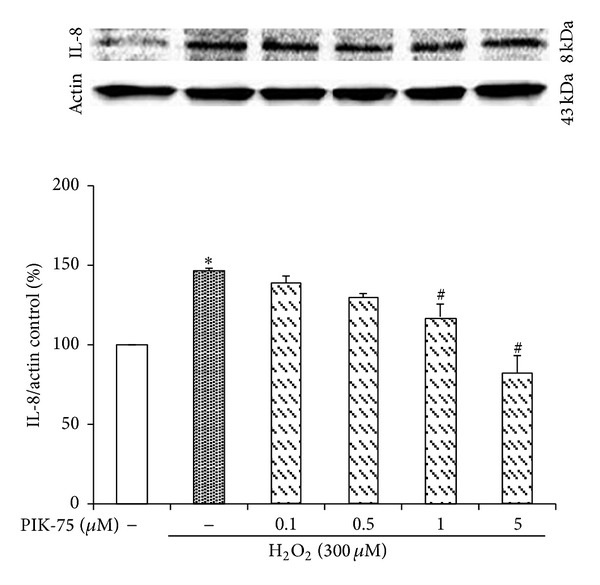
Effect of PIK-75 on IL-8 expression in feline EECs stimulated by H_2_O_2_. Serum-starved EECs were preincubated in the presence of PIK-75. EECs were then stimulated with H_2_O_2_ (300 *μ*M, 6 hours). The expression of IL-8 was estimated by Western blot analysis. Data are expressed as means ± S.E of four experiments (Student's* t*-test; *P < 0.05 versus control, ^#^ *P* < 0.05 versus H_2_O_2_ alone).

The cells were pretreated with PIK-75 at the indicated concentrations and exposed to 300 *μ*M hydrogen peroxide, and then IL-8 expression was measured by Western blot to examine whether PIK-75 downregulates IL-8 expression in cultured EECs (Figure 7). The expression of IL-8 was upregulated when treated with 300 *μ*M hydrogen peroxide for 6 hours. The cells were pretreated with different doses of PIK-75 (0.1, 0.5, 1, and 5 *μ*M) for 1 hour to examine the effect of PIK-75 on H_2_O_2_-induced IL-8 expression.PIK-75 inhibited the IL-8 expression in a dose-dependent manner.

### 3.8. Hydrogen Peroxide-Induced IL-6 Release Is Reduced by PIK-75 Treatment

**Figure 8:**
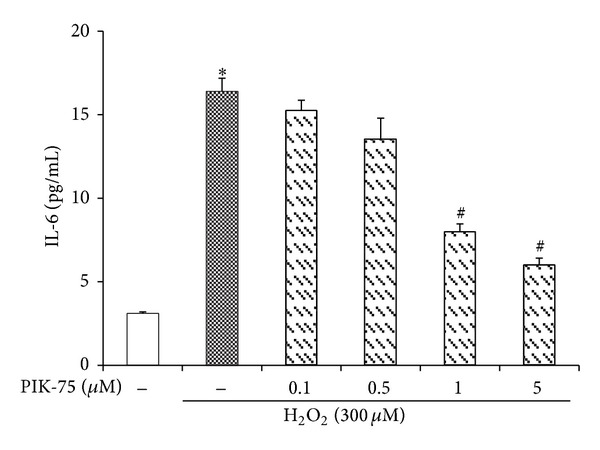
Effect of PIK-75 on IL-6 release in feline EECs stimulated by H_2_O_2_. Serum-starved EECs were preincubated in the presence of PIK-75. EECs were then stimulated with H_2_O_2_ (300 *μ*M, 6 hours). The production of IL-6 was measured in supernatants by ELISA. Data are expressed as means ± S.E of four experiments (Student's* t*-test; *P < 0.05 versus control, ^#^ *P* < 0.05 versus H_2_O_2_ alone).

The inhibitory effect of PIK-75 in the hydrogen peroxide-induced IL-6 release was determined using IL-6 ELISA kit (Figure 8). The data showed that the treatment of cultured EECs with hydrogen peroxide caused a significant increase in the IL-6 production. However, the IL-6 production level was reduced in a dose-dependent manner when the EECs were treated with PIK-75.

## 4. Discussion

PI3K is an important regulator of inflammations and its isoforms have been studied as therapeutic targets [34]. It seems to be related with esophageal diseases such as Barrett's esophagus and esophageal cancer [7, 35]. In this study, we investigated the expression of PI3K isoforms in feline EECs and their role in the mediation of esophageal inflammations.

In the present study, hydrogen peroxide, as one type of ROS that is elevated in GERD, exhibited significant cytotoxicity and decreased the cell viability in EECs. This cytotoxicity of hydrogen peroxide seemed to be related with its ability to induce IL-1*β*, IL-8, and IL-6, suggesting a role for classic stress signaling pathways during esophagitis and GERD [36–38]. It is consistent with the effect of hydrogen peroxide in the esophagus, inducing IL-1*β* and IL-6 [39].

In this study, p110α isoform was the mostly predominant PI3K isoform in EECs when it came to the inflammatory responses induced by hydrogen peroxide. p110α showed the biggest expression difference when hydrogen peroxide was added. Although the expression of p85α, p85*β*, and p55*γ* was at high level in normal condition, it was not significantly changed after the treatment with hydrogen peroxide. For those reasons, we assume p110α may play an essential role in esophageal inflammation. This result is consistent in the distribution of human intestinal epithelial crypt (HIEC) cells meaning that p110α is a predominant PI3K isoform [40].

In this study, PIK-75 was used as a preferential p110α PI3K inhibitor [41] to confirm the role of p110α in esophageal epithelial inflammation. PIK-75 has been studied and showed its efficacy in many different cells. For instance, it regulates the secretion of intestinal peptide neurotensin in the small bowel [42]. Furthermore, the delivery system of PIK-75 was investigated due to an increasing interest in PIK-75 [43, 44].

The inhibition of p110α was confirmed due to an expression examination of p-Akt because p110α-positive cases were also positive for p-Akt [45]. The expression of p-Akt was decreased depending on the concentration of PIK-75 if p-Akt was treated with hydrogen peroxide. Moreover, PIK-75 downregulated the production of IL-1*β*, IL-6, and IL-8 as proinflammatory cytokines when the cells were cotreated with hydrogen peroxide, while the production of IL-1*β*, IL-6, and IL-8 was increased when it was only treated with hydrogen peroxide.

The IL-8 expression was increased as an esophagitis inducer in human EECs if it was treated with bile acids [46]. Patients with reflux esophagitis have high levels of IL-8 and IL-1*β* and patients with adenocarcinoma show markedly elevated levels of IL-8 and IL-1*β* [47]. The expression of IL-6 and IL-8 is elevated in the esophageal mucosa of patients with GERD [48]. For those reasons, it is important that PIK-75 decreases the production of IL-1*β*, IL-6, and IL-8 in feline EECs.

Furthermore, the requirement for PI3K/Akt in the induction of IL-6 genes in response to IL-1*β* is revealed [49, 50]. Therefore, an upregulation of IL-1*β* may enhance the PI3K pathway mediating inflammation.

The present study data indicate an engagement of p110α in the inflammation suppression in EECs as well as a role as an important mediator in the signaling cascade that leads to an initiation of the inflammatory response.

Although PI3K is a good target for the development of anti-inflammation drugs, PI3K inhibitors have some limitations. PI3K inhibitors are associated with toxicities resulting from off-target effects that cannot not be fully explained yet [51]. Targeting a single isoform would have fewer side effects and avoid the toxicity in immune system. However, it remains a challenge to identify the optimal concentration and exposure time for therapeutic uses and a more specific signaling in EECs. Furthermore, full investigation of the expression and the roles of PI3K isoforms in many different cells could prevent side effects when inhibitors are used as drugs.

As a result of this study we found that hydrogen peroxide, which is highly expressed in esophagitis, activates PI3K signaling and that p110α plays an important role in esophageal inflammation. By hydrogen peroxide increased proinflammatory cytokines were reduced by the treatment of PIK-75, a p110α inhibitor. Therefore, these data suggest that PIK-75 has an anti-inflammatory effect in feline EECs.
